# Post-Thymectomy Autoimmune Flare-Up With New-Onset Type 1 Diabetes Mellitus

**DOI:** 10.1210/jcemcr/luae039

**Published:** 2024-03-22

**Authors:** Tareq Al-Bkoor, Fateen Ata, Ammara Bint I Bilal, Mohammed Abdulgayoom, Honar Cherif, Haval Surchi

**Affiliations:** Department of Internal Medicine, Hamad Medical Corporation, Doha, Qatar; Department of Endocrinology and Metabolism, Hamad Medical Corporation, Doha, Qatar; Department of Radiology, Hamad Medical Corporation, Doha, Qatar; Department of Hematology, Hamad Medical Corporation, Doha, Qatar; Department of Hematology, Hamad Medical Corporation, Doha, Qatar; Department of Endocrinology and Metabolism, Hamad Medical Corporation, Doha, Qatar

**Keywords:** thymectomy, type 1 diabetes, diabetes mellitus, autoimmune diabetes, post-thymectomy autoimmune flare

## Abstract

The thymus gland aids in the maturation of the immune system. An overactive or malfunctioning thymus gland, as seen in thymomas, can lead to disrupted immune systems. Thymectomy, the usual treatment, can paradoxically lead to further derangements in the immune system, leading to new autoimmune disorders. Most of these reported disorders are rheumatological. Except preclinical studies, there are no reported cases of autoimmune diabetes post-thymectomy. A 25-year-old woman who had malignant thymoma underwent chemotherapy, followed by thymectomy and radiotherapy. She developed autoimmune diabetes mellitus (AID) approximately 1 year post-thymectomy, evident from raised glycated hemoglobin, anti-glutamic acid decarboxylase (GAD) antibodies, ineffectiveness of oral glucose-lowering agents, and positive response to insulin. AID can occur after thymectomy, as evidenced by animal studies and this case report. Whether these patients would have long-term outcomes and control of diabetes differently than classic type 1 diabetes mellitus (T1D) is uncertain. Further research is needed to prove causality between thymectomy and diabetes.

## Introduction

The thymus is a lymphoid organ that plays a critical role in the immune system, particularly in the development and maturation of T cells, which are essential for adaptive immunity (1). T-cell precursors from the bone marrow differentiate into various types of functional T cells via the thymus, mainly in the younger years of life (2). A thymoma is a tumor originating from the thymus's epithelial cells. It is a rare tumor, representing up to 1.2% of all malignancies. Benign and malignant thymomas (MT) can alter the standard architecture and function of the thymus, leading to immunological abnormalities. Though encapsulated and less aggressive, benign thymomas can still affect the immune system and are often associated with autoimmune diseases such as myasthenia gravis. MT, on the other hand, are more invasive and can lead to more severe immunological dysfunctions, as well as a much higher mortality rate (3).

Thymectomy, a cornerstone of benign thymoma or MT, can lead to immune-related complications after thymectomy (4). These include systemic lupus erythematosus, Sjogren syndrome, rheumatoid arthritis, Hashimoto thyroiditis, systemic vasculitis, and pemphigus vulgaris in humans and autoimmune gastritis in mice (5-7). Hyperfunctioning of the thymus gland can lead to the proliferation of both the stimulator and suppressor lymphocytes. Resultant thymectomy can alter the autoimmune status of patients based on the type of cells removed (8). Animal studies have indicated a connection between thymectomy and the onset of type 1 diabetes mellitus (T1D). However, there is a lack of clinical studies to confirm this association in humans (9). To the best of our knowledge, no previous case of T1D following thymectomy and radiation has been documented.

## Case Presentation

A 25-year-old Jordanian woman was admitted to the hospital with hyperglycemia. She did not have any symptoms (including polydipsia, polyphagia, abdominal pain, or any other symptoms suggestive of a hyperglycemia state) and was incidentally found to have a glucose reading of 22.5 mmol/L (405 mg/dL) (reference range, 3.9-7.8 mmol/L; 70-140 mg/dL). The patient had a known diagnosis of MT, managed with chemotherapy, radiotherapy, and thymectomy. She had pure red cell aplasia post-thymectomy, was on corticosteroids. The initial impression was probable steroid-induced hyperglycemia. The patient's body mass index (BMI) at presentation was 18.1 kg/m^2^. She did not report a family history of diabetes or other autoimmune disorders.

**Figure 1.**
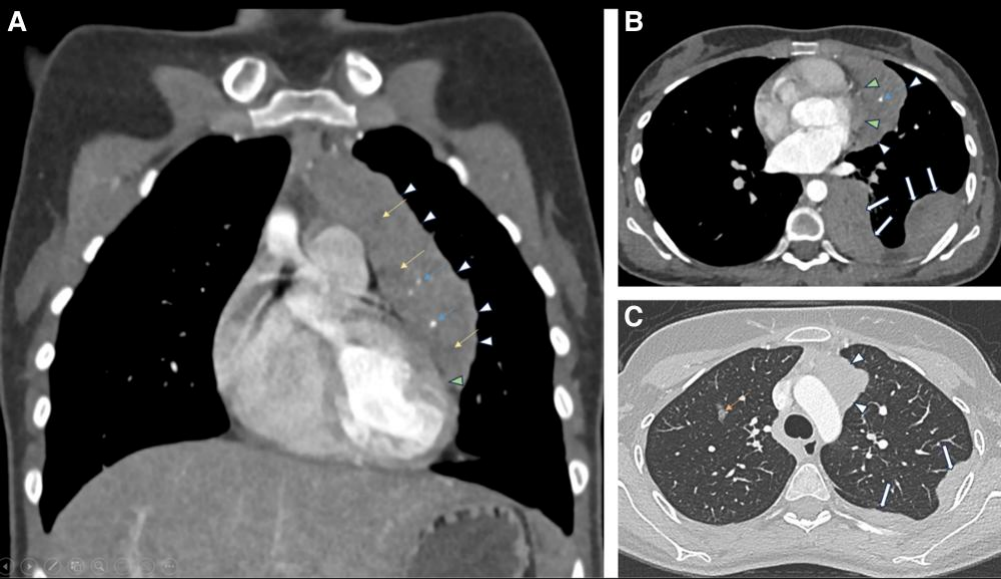
Selected images from contrast-enhanced chest CT scan demonstrated a large lobulated left anterior mediastinal mass (white arrowheads) with intrinsic coarse calcifications (blue arrows) and hypodense areas of necrosis (yellow arrows) in the soft tissue window coronal (A) and axial (B) reconstructions. There is a left pericardial invasion (green arrowheads). Multifocal left pleural nodules and masses (white solid arrows) representing solid metastases are also seen. Lung window axial reconstruction (C) reveals right lung upper lobe parenchymal ground glass nodules (orange arrow) in addition to the mediastinal mass (white arrowheads) and pleural deposits (white solid arrows). Features are consistent with invasive thymoma.

**Figure 2.**
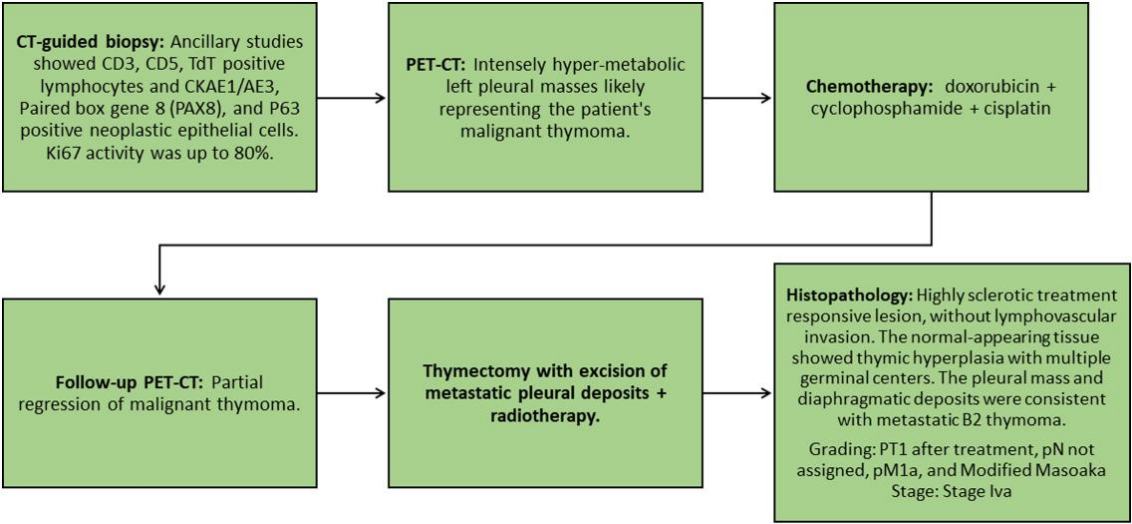
Flow diagram of diagnostic workup and management of patient's malignant thymoma. Abbreviations: CT, computed tomography; PET, positron emission tomography; TDT, terminal deoxynucleotidyl transferase.

The patient's thymoma was diagnosed 7 years ago and was limited to anterior mediastinum. She did not undergo further workup and was lost to follow-up. Approximately 1 year preceding the onset of hyperglycemia, the patient presented to the primary healthcare clinic with a productive cough and fever. The review of systems and physical examination were otherwise unremarkable. Chest x-ray was done to rule out lower respiratory tract infection, which showed the previously known mediastinal mass. However, the x-ray revealed left pleural nodules and masses. A computed tomography (CT) scan of the thorax was done, which revealed pericardial and pleural invasion of the mediastinal mass, denoting invasive MT (Fig. 1). Further workup confirming the diagnosis and the management of MT is shown in Fig. 2.

The patient's treatment was complicated with pure red cell aplasia. She had a remote history of iron deficiency anemia, hemoglobin (Hb) (9.6 gm/dL) (96 g/L) (reference range, 12-15 gm/dL; 120-150 gm/L) with serum iron 5 umol/L (reference range, 6-35 umol/L) and iron saturation 7% (reference range, 15-45%). However, the patient received iron supplementation, and her Hb has been stable over the years, including the pre-thymectomy phase. Post-thymectomy Hb dropped to a nadir of 6.6 mg/dL (66 g/L), with bone marrow biopsy confirmation of pure red cell aplasia.

## Diagnostic Assessment

**Figure 3.**
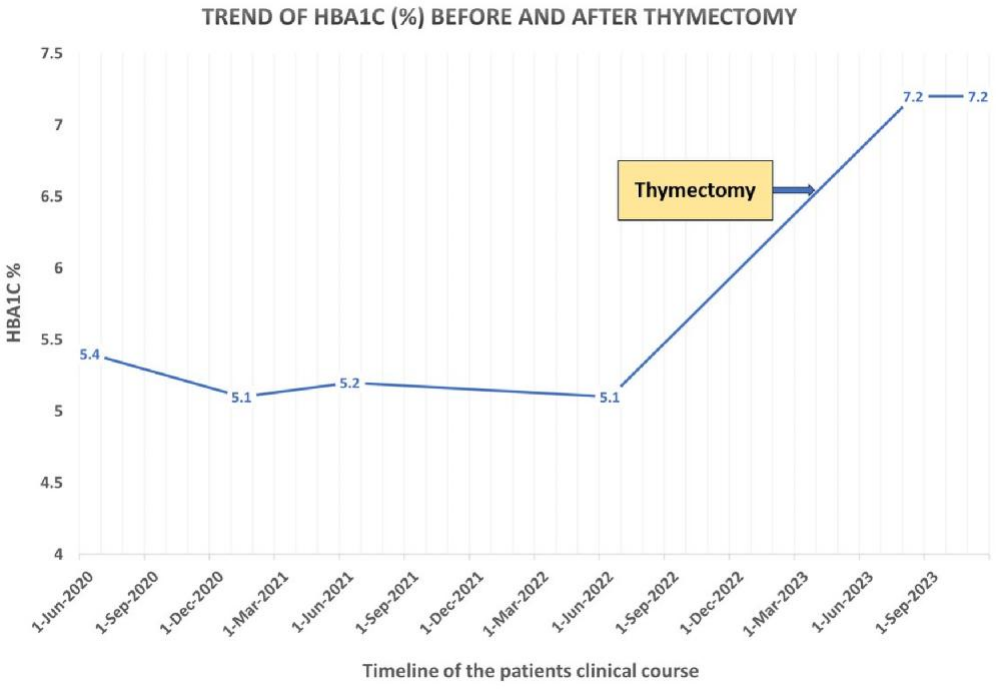
The trend of HbA1c (%) for the patient before and after thymectomy.

**Figure 4.**
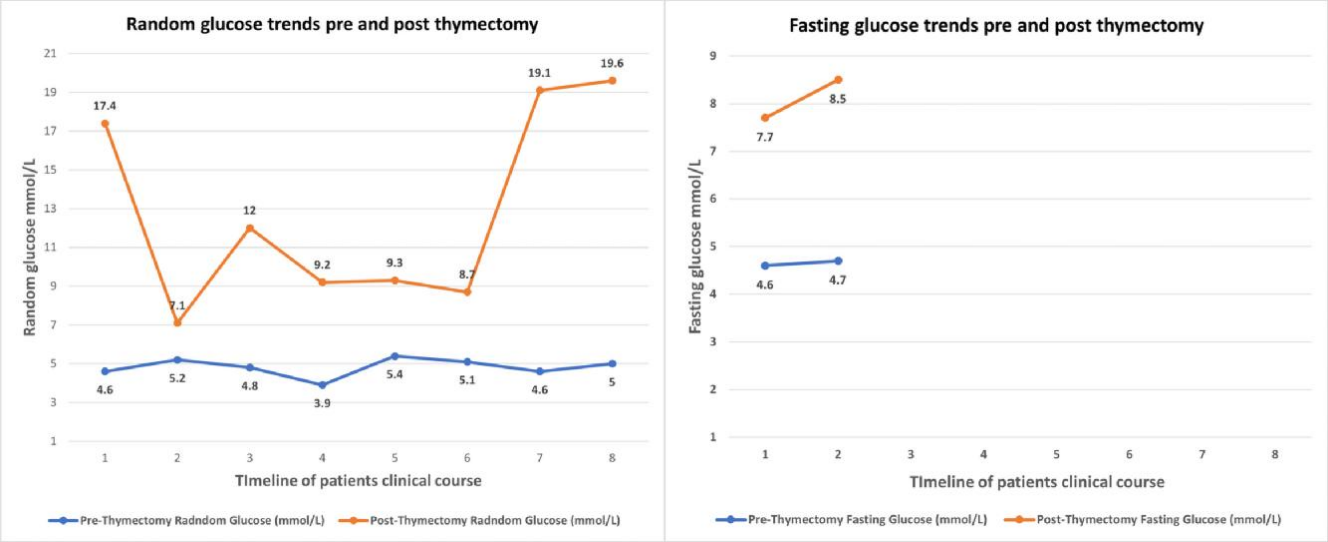
The trend of fasting glucose (mmol/L) and random blood glucose (mmol/L) before and after thymectomy.

Workup showed glycated hemoglobin (HbA1c): 7.2% (55.2 mmol/mol) (reference, < 5.7%, <39 mmol/mol), random glucose: 17.4 mmol/L (313.2 mg/dL) (reference range, 3.9-7.8 mmol/L; 70-140 mg/dL), fasting glucose: 22.5 mmol/L (405 mg/dL) (reference range, 3.9-5.5 mmol/L; 70-99 mg/dL), glucosuria 4+, and ketonuria 3+. However, serum ketones, bicarbonate, and pH were normal, ruling out diabetic ketoacidosis. Further workup revealed strongly positive anti-GAD antibodies (> 2000 IU/mL) (reference range, 0-10 IU/mL), with a low C-peptide of 0.58 ng/mL (0.20 nmol/L) (reference range, 1.1-4.4 ng/mL; 0.36-1.45 nmol/L). The corresponding fasting glucose at the time of C-peptide extraction was 8.5 mmol/L (153 mg/dL). Based on the laboratory investigations, a diagnosis of T1D was made, ruling out the initial impression of steroid-induced hyperglycemia. The patient had previous HbA1c values in the normal range before thymectomy and well after diagnosis of thymoma (Fig. 3). Pre-thymectomy fasting glucose and random glucose levels were also checked on multiple occasions over the years. They were within the normal limits, whereas post-thymectomy values established the diagnosis of diabetes (Fig. 4). Apart from a negative antinuclear antibody (ANA), no other autoimmune test was done before thymectomy (including pre-thymectomy anti-GAD antibodies).

## Treatment

The hyperglycemia was managed inpatient with regular insulin 6 units, 3 times daily. Early identification of the insulin-deficient state with rising urine ketones played a crucial role in preventing diabetic ketoacidosis. The patient was reluctant to take insulin post-discharge and was given a trial of gliclazide 60 mg once daily, without effect. Due to persistent hyperglycemia and with the confirmation of T1D diagnosis, she was counseled on appropriate treatment. She was started on insulin Toujeo 10 units and insulin Aspart 8 thrice daily before meals.

## Outcome and Follow-Up

The patient's HbA1c was stable at 7.2% without any symptoms at her 3-month follow-up. Diabetes educators saw her in between follow-ups, and her insulin Toujeo was increased to 12 units due to uncontrolled fasting glucose readings (> 11.1 mmol/L (> 200 mg/dL). She was taking prednisolone oral tablet 30 mg once daily. The patient subsequently tapered off prednisolone 1 month later. She currently takes 10 units of Toujeo daily and 3 to 6 units of Aspart before each meal. Her fasting readings remain between 5 and 7.2 mmol/L (90-130 mg/dL). She does not check postprandial glucose readings.

## Discussion

Autoimmune diabetes (AID) results from the immune system erroneously attacking β-cell antigens, leading to inflammation and β-cell destruction (10). The process starts with the immune system producing autoantibodies and dendritic cells presenting β-cell antigens to T cells. Autoimmune responses occur when autoreactive T cells, evading negative selection in the thymus, activate cytotoxic T and B cells against self-antigens, causing AID (11). The thymus is specialized in fostering the development of diverse and functional T cells that exhibit self-tolerance, with thymopoiesis regulated through distinct checkpoints (1, 12). Cortical thymic epithelial cells (cTECs) and medullary thymic epithelial cells (mTECs) promote positive and negative selection, respectively, ensuring the elimination of autoreactive T cells and the production of a diverse spectrum of T cells and regulatory T cells (Tregs) to prevent autoimmunity (13). This process results in a comprehensive repertoire of peripheral naïve T cells with diverse recognition capabilities against various pathogens and subsets of regulatory T cells (Tregs) that control excessive immune responses and autoimmunity (1).

The precise mechanisms through which thymoma triggers autoreactivity are yet to be fully elucidated. However, the association between thymoma and autoimmune diseases appears well-established and not merely coincidental (3). Removing thymoma has shown therapeutic benefits in patients with specific autoimmune conditions. The primary hypothesis explaining thymoma-related autoimmunity posits that the thymus's compromised self-tolerance, resulting from tumor growth, creates a favorable environment for the development of autoimmune diseases (3). Other than surgical complications, long-term complications have also been reported after thymectomy. These include serious health issues such as cancers and autoimmune disorders (4). A recent comprehensive analysis of the Mass General Brigham Research patient data registry has revealed elevated all-cause mortality (8.1% vs 2.8%; relative risk [RR] 2.9; 95% CI 1.7-4.8) and an increased risk of cancer (7.4% vs 3.7%; RR 2.0; 95% CI 1.3-3.2) in individuals who underwent thymectomy for various indications and approaches when compared to a matched control population (those undergoing cardiac surgery without thymectomy). Additionally, the risk of autoimmune disease was found to be higher in thymectomy cases (12.3% vs 7.9%; RR 1.5; 95% CI 1.02-2.2), notably when patients with preoperative infection, cancer, or autoimmune disease were excluded from the analysis (4). The enigmatic paradox of autoimmune diseases associated with thymoma and post-thymectomy remains an area of incomplete understanding, warranting further scientific exploration. Various autoimmune diseases have been reported post-thymectomy in the literature. The data from these studies have been summarized in Table 1. The understanding of thymectomy's impact on autoimmune diseases expanded with a nationwide study in Taiwan encompassing 2250 thymectomized patients. Compared to controls, the patients who had undergone thymectomy exhibited a 2.68 times higher overall incidence rate of any autoimmune disease (26). The timing of thymus removal seems to play a pivotal role in immunological outcomes, influencing the balance of autoreactive and regulatory T cells. Thymectomized patients often display mild T-cell lymphopenia linked to B-cell hyperreactivity and autoantibody titers, potentially contributing to autoimmune diseases (26).

**Table 1.** Summary of clinical course and outcomes of patients with post-thymectomy autoimmune disorders

Authors, year	N	Meanage	Sex	Thymic disorder	Prior AI disease	New AI disease	RT	Thymectomy to AI disease time	Outcome
Isasi et al, 2020 (14)	3	37.6	1. F 2. F 3. F	1. Thymic hyperplasia 2. Thymic hyperplasia 3. Thymic hyperplasia	1. OB-MG. 2. GMG 3. BMG	1. SLE and APS 2. SLE 3. SLE	No	6 years 7 years 3 years	NM
Wickemeyer et al, 2014 (15)	1	59	M	Microinvasive thymoma	No	SID	Yes	2 weeks	Chronic stable ID
Furukawa et al, 2018 (16)	1	74	F	Type AB thymoma	Lichen Planus	Good’s syndrome	No	19 months	Died of repeated infections
Miskovic et al, 2015 (6)	1	48	F	NM	GMG	SLE and secondary APS	No	28 years	NM
Gonlugur et al, 2006 (17)	1	53	M	Mixed type (lymphoepithelial) thymoma	No	MG	No	1 month	Died due to sepsis
Omar et al, 2010 (18)	1	13	F	NM	MG	SLE	No	3 years	NM
Gurowich et al, 2021 (19)	1	82	F	Type AB thymoma, Masaoka-Koga stage 1	No	MG	No	3 months	Diplopia and dysarthria were resolved over time
Kang et al, 2007 (20)	1	39	M	Noninvasive medullary thymoma	No	MG	No	3 years	Stable on pyridostigmine at 2-year follow-up
Kobza et al, 2022 (21)	1	52	F	NM	MG	MCD	No	10 years	Awaiting autologous HSCT for treatment of both refractory MG and MCD
Sawamura et al, 2017 (22)	1	65	F	Type AB thymoma	No	SLE, MG, chronic thyroiditis, and pemphigus foliaceus	No	1 year	Stable on 15 mg/day prednisolone
Neokleous et al, 2022 (23)	1	46	F	NM	MG	graft vs host-like syndrome associated with pure white cell aplasia	No	8 years	Died of multiorgan failure
Park et al, 2003 (24)	1	60	F	Lymphocytic thymoma	No	Aplastic anemia	No	16 months	Stable after treatment
Liu et al, 2013 (25)	1	80	F	Type AB minimally invasive thymoma	No	Good’s syndrome	No	5 years	Became afebrile, and the diarrhea settled 2 days after receiving IVIG

Abbreviations: AI, autoimmune; APS, antiphospholipid syndrome; B, bulbar; F, female; GMG, generalized myasthenia gravis; HSCT, hematopoietic stem cell transplant; IVIG, intravenous immunoglobulins; ID, immunodeficiency; M, male; MCD, mixed connective tissue disease; MG, myasthenia gravis; NM, not mentioned; OB, oculo-bulbar; RT, radiotherapy; SID, severe immunodeficiency; SLE, systemic lupus erythematosus.

Evaluation of AID post-thymectomy is limited to animal studies. In a study on bio-breeding diabetes-resistant (BBDR) rats, Ramanathan et al induced T1D through thymectomy and sublethal irradiation (9). The study revealed an age-dependent susceptibility and the role of T-cell subsets. The authors argued in favor of an autoimmune basis marked by insulitis and characteristic diabetes symptoms in the rats after thymectomy (9). Transfer of T cells from diabetic rats to nondiabetic recipients establishes T-cell mediation, with syngeneic CD45RC−, CD4+ T cells preventing diabetes. In the study, the propensity for thymectomy-induced diabetes was determined to be genetically controlled, as only non-lymphopenic specimens genetically akin to the bio-breeding diabetes-prone (BBDP) strain manifested diabetic symptoms following the intervention. This hints towards a hereditary vulnerability within this rat strain, representing a genetic inclination towards diabetes in response to thymectomy and radiation. This research illuminates the intricate interplay of immune alterations and genetics in T1D development following thymectomy and irradiation (9). Another extensive nationwide retrospective review of 5664 patients who underwent thymectomy in the early years of life (before age 5) reviewed long-term outcomes in the patients compared to controls. In this study, the authors compared hazard ratios (HR) for the development of various disorders in patients and showed an HR of 3.16 (1.08-9.21) for the development of T1D in thymectomized patients compared to surgical controls (patients who underwent early-life cardiac surgeries without thymectomy). However, the study could not show a statistically significant risk of T1D development in thymectomized patients compared to the general population (HR 1.13 [0.76-1.67]) (27). A comprehensive review by Vincent Geenen underscores the role of thymic dysfunction in diabetogenic autoimmunity (28). Aberrations in the pivotal function of the thymus in the maturation and education of T lymphocytes can result in the emergence of autoreactive T cells, which may precipitate the pathogenesis of AID. Historical and experimental evidence supports the hypothesis, including neonatal thymectomy studies and transplantation experiments. Genetic factors, such as IDDM2, linked to lower INS transcripts in the thymus, enhance our understanding of the complex interaction between thymic function and autoimmune diseases like T1D (28).

## Learning Points

- AID can manifest post-thymectomy, supported by findings from animal studies and this report.
- The long-term prognosis of AID post-thymectomy and how it may diverge from the typical course of classical T1D remains uncertain.
- Although definitive evidence linking thymectomy to the etiology of AID has not been established, various new autoimmune diseases arising after thymectomy and initial observations regarding diabetes indicate a potential correlation.

## Data Availability

Data sharing is not applicable to this article as no datasets were generated or analyzed during the current study.

## Abbreviations

AID: autoimmune diabetes mellitus
GAD: glutamic acid decarboxylase
Hb: hemoglobin
HbA1c: glycated hemoglobin
HR: hazard ratio
MT: malignant thymoma
RR: relative risk
T1D: type 1 diabetes mellitus
